# Atoms

**DOI:** 10.1136/archdischild-2024-327339

**Published:** 2024-05-17

**Authors:** Nick Brown

**Affiliations:** 1 Department of Child and Women's Health (KBH), Uppsala University, Uppsala, Sweden; 2 Department of Paediatrics, Länssjukhuset Gävle-Sandviken, Gävle, Sweden; 3 Aga Khan University, Karachi, Pakistan

**Keywords:** Adolescent Health, Allergy and Immunology, Analgesia

## Mixed messages

To use the emergency department vernacular, this family was a ‘frequent flyer’. If you took the time to check the entries from 54/55 one of the three children would attend on a near weekly basis. Closer scrutiny would also have shown that most attendances were in the mid to late evening. It would have taken little extra effort to conclude that the symptoms were rarely distressing for the children: an URI, a sprained ankle, a barely discernible scratch from an errant kite in Central. Never more than an advice and acetaminophen job. Of more concern (at least so was said by the interns retrospectively) was that it was always the mother, E, in her early 20 s who came, her husband, stolidly, ‘at home entertaining the others with cookies and the latest Disney cartoons on the colour TV inherited from his own parents as a wedding gift’. Irrespective of the time of year, she was fastidiously dressed. Achingly vogue, upturned collar ruffs, silk long sleeves, they clearly weren’t short of money. Irrespective of the time of year though, she looked anxious and distracted. Nothing would have raised any suspicion beyond the gossip level, had it not been for the visit in the late Autumn of ’56. She’d clearly left her block of flats quickly, her usual attention to detail neglected with the result that her usually hidden limbs and neck were for the first time, unexposed. Her daughter had toothache on that occasion, but the staff’s only concern, the multiple bruises and bite marks adorning E’s now unmasked skin. When the officers entered the flat just a couple of hours later, a luridly-coloured Mickey Mouse was passing the baton to Donald Duck, the husband (clearly having gained a few pounds since the time the framed picture, prominent on the living room wall, from the Korean conflict was taken) slurring but still ambulatory, a half empty bottle of Jack Daniels in his pocket, the children asleep, fully clad on the kitchen floor.

## Pea soup(er) for lunch?

What we knew in 2023. We knew that air pollution, particularly in the form of PM 2.5, PM 10, NO_2_, SO_2_, ozone and CO_2_ are noxious and that the WHO had guidelines on acceptable levels of these ubiquitous toxins. We knew that children spend a large proportion of their waking hours in school and therefore, that an unsafe environment during the day would have major implications for cumulative exposure. We knew how eager policymakers were to stress the importance of legislation. Knowing all this, new information in 2024 is at the very least, discomfiting. Using fully accessible data from the UK Central Office of Public Interest on levels of pollution at proposed new primary school sites, Yasmin Mahfouz and colleagues estimates of the future environment for today’s toddlers, are more than unsettling. Of those proposed schools with confirmed locations, 86% exceeded all three WHO Air Quality Guidance targets, and all exceeded least one. Nationally, close to 80% were in the 60th or greater pollution percentile figures, London ‘scoring’ particularly high with a median pollution percentile of 90. Shocking? Of course. Worse still, is the lack of mandatory assessment of air pollution when new sites are proposed… Where did the chain of responsibility break down? ***See page 483***

## Approaches to Rome

We’re all, of course, entitled to use treatments known to be beneficial in relevant situations and, it’s no surprise therefore that there is a degree of heterogeneity at individual level. If authoritative guidance is also mixed, then variation is magnified. In the Paediatric Emergency Medicine section, Simon Craig and colleagues from the Paediatric Emergency Research Network (PERN) group report an assessment of guidance currently in use for acute asthma, identifying 158 references globally. Though there is substantial overlap between recommended drugs, the stage at which they are introduced, and rate of escalation varies widely, a reflection perhaps of interpretation of data (evidence-based) and personal preference from type and delivery of respiratory support to parenteral drugs. So, in essence, the same child would receive geography- dependent care depending on where she presents. The authors’ suggestion of collaboration with the Global Initiative for Asthma (GINA) group seems eminently sensible. ***See page 468***

### What is her status?

We know the drill: a seizing child, unresponsive to ADC and first line drugs, receives escalating IV treatment, the anaesthetists are informed and the call to the paediatric intensive care unit (PICU) is made. Those still resistant, need to be sedated and intubated, in short, undergo rapid sequence induction (RSI). This process, inevitably, makes seizure assessment more difficult as clinical signs must be replaced by ambulatory (or full if one has the luxury) EEG monitoring. Philip Knight and a national PICU group assess what happens next and the implications for transfer services. In short, they found that with active dialogue between teams, a high proportion were successfully extubated and subsequently managed at the local hospital and physical transfer avoided, a win-win providing there is active dialogue between teams. ***See page 476***

## Menage a…

The literature demonstrating the associations between internet use and (to name but a few) obesity, mental health and sleep disturbance is now so vast that dissent near impossible. When screen-derived advice starts to impinge on clinical decision-making, though, the issues become both more palpable and less palatable. Clare Delaney and colleagues’ review and Dominic Wilkinson’s linked editorial, describe the situation (to many of us now familiar) when parents have sought advice online and support through social media and the tensions this generates. There are a number of mitigation strategies, but as the flags in the recent Nuffield report emphasise, the scale of potential collateral damage to everyone involved cannot be overstated. ***See pages 458 and 453***

